# Pulmonary embolism mimicking acute myocardial infarction: a case report and review of literature

**DOI:** 10.11604/pamj.2019.33.275.18517

**Published:** 2019-07-30

**Authors:** Saida Zelfani, Hela Manai, Saoussen Laabidi, Abir Wahabi, Sara Akeri, Mounir Daghfous

**Affiliations:** 1 Pre Hospital Emergency Department (SAMU 01), Emergency Medical Help Center of Tunis, Tunis, Tunisia

**Keywords:** Myocardial infarction, pulmonary thromboembolism, electrocardiography

## Abstract

The diagnosis of pulmonary thromboembolism (PTE) with changes shown by electrocardiography (ECG) is a challenge in the clinical practice due to rare pathognomonic findings. We report the case of a 37-year old woman managed in out of hospital sitting for a chest pain. Electrocardiogram was suggestive of antero-septal acute myocardial infarction (AMI). Catheterization revealed non occlusive coronary disease. Transthoracic echocardiography showed an elevated pulmonary and right heart pressures. Computed tomography pulmonary angiography confirmed the diagnosis of bilateral pulmonary embolism. PTE with ECG changes should be considered in the differential diagnosis of AMI, particularly in young patients with chest pain and ST segment elevation suggestive of acute coronary syndrome.

## Introduction

The ST segment elevation represents common electric sign of acute transmural ischemia caused by an occlusion of an epicardial coronary artery by a blood clot. Especially in pre hospital care and without other investigations, urgent therapy for patients with chest pain and ST elevation must be considered to reanalyze the occluded artery by percutaneous coronary intervention or fibrinolysis when cat lab is unavailable or far away. Symptoms of pulmonary thromboembolism (PTE) and acute myocardial infarction (AMI) can be similar, including acute dyspnea, chest pain, syncope and palpitations. Physical examination is nonspecific and cannot reliably distinguish these two diagnoses. Electrocardiogram (ECG) may be helpful for the diagnosis of PE but its limited by his sensitivity and specificity [1-3]. Although several ECG changes can be observed in the acute phase of PTE, ST segment elevation is a rare finding [4-6]. We report a case of woman who had dynamic ST segment elevation suggestive of antero-septal AMI that proved to be bilateral PTE.

## Patient and observation

**Figure 1:**
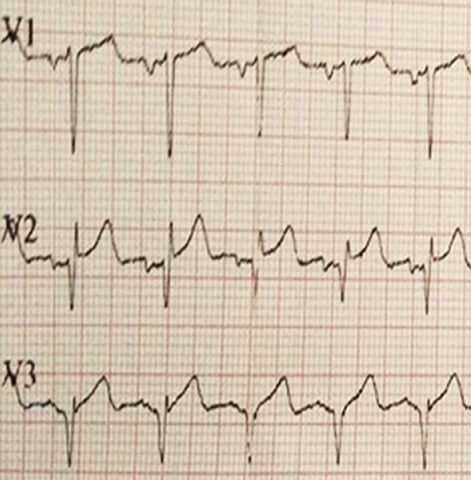
Electrocardiogram shows ST-segment elevation of 2 mm in leads V2 and V3

**Figure 2:**
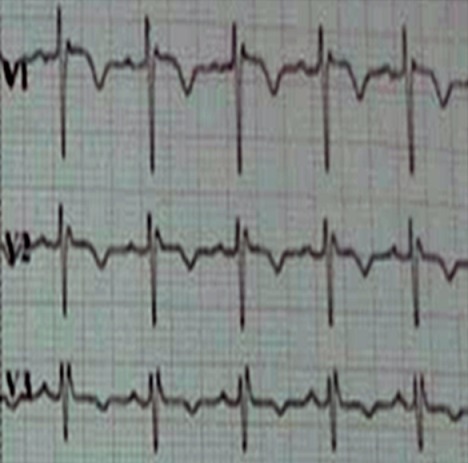
Electrocardiogram shows a right bundle branch block (RBBB) in leads V1, V2 and V3

**Figure 3:**
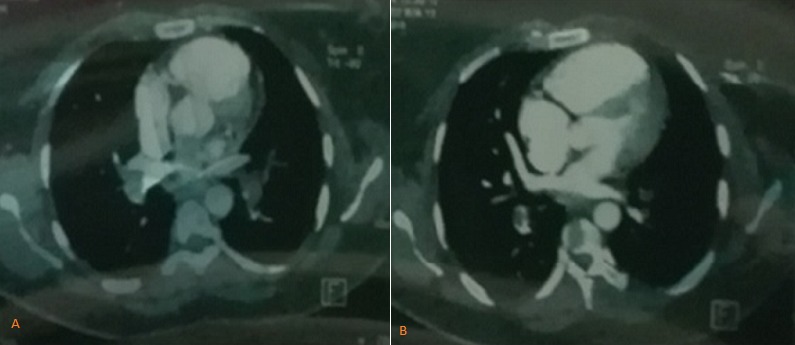
Chest CT scan showing proximal pulmonary embolism (A) and dilated right ventricle (B)

A 37-year-old woman, without past medical history, presented to emergency room in primary center, complaining of chest pain, acute coronary syndrome was suspected. Our emergency medical system received call for this patient and activated pre hospital emergency team for transfer. The patient suffered from continuous acute two hours before our intervention. She doesn't have previous history of similar episode. No previous history of coronary artery disease, peripheral vascular disease, stroke, malignancy, or venous thromboembolism was reported. There was no family history of thromboembolic disease. Physical examination revealed: a regular pulse rate 110 beats/min, blood pressure was 100/65mmHg, respiratory rate wa 20 breaths/min, oxygen saturation was 95% at room air and 99% with 2l/min oxygen via nasal canula and temperature was 37°C. Cardiac auscultation was normal. There were no congestive neck veins. An initial ECG showed a sinus rhythm, an ST segment elevation of 2 mm in V2 and V3 without other anomalies (Figure 1). The initial diagnosis of antero-septal AMI was established. After initiating treatment by Aspirin (250 mg), Clopidogrel (300 mg) and intravenous heparin, the patient was transferred to cat lab. Twenty minutes later a second ECG showed a right bundle branch block (RBBB) with disappearance of ST segment elevation on precordial leads (Figure 2). Coronary angiography performed forty five minutes later, however showed normal coronary arteries without stenosis. Transthoracic echocardiography (TTE) revealed right ventricular dilatation and elevated pulmonary arterial pressure (HTAP = 60 mmHg). Computed tomography pulmonary angiography concluded to proximal bilateral pulmonary embolism with dilated right ventricle and paradoxical septum (Figure 3). A Doppler ultrasound of the lower extremities did not show any finding compatible with deep venous thrombosis. An intravenous heparin was initiated with good outcome. An etiological investigation of thromboembolic disease demonstrated the presence of deficiency of protein C. Oral anticoagulation was initiated before discharge from the hospital. The patient was doing well at 3 months of follow-up.

## Discussion

The ECG still has a major role in diagnosing and triage of patients presenting with chest pain [7]. The current American college of cardiology/American heart association (ACC/AHA) guidelines for STEMI recommend that patients with suggestive symptoms of myocardial ischemia who have ST segment elevation at the J point (in 2 contiguous leads or more of 0.2 mV or more in males or 0.15 mV or more in women in leads V2V3 and/o of 0.1 mV or more in all other leads in threshold) should undergo immediate reperfusion therapy [8]. Otto found that 63 patients among 123 (59%) with chest pain and ST elevation in pre hospital care had diagnosis rather than AMI [9]. In another study, Brady found that 157 patients among 212 (74%) presenting with chest pain had elevation of the ST segment due to non ischemic etiologies. This condition is challenging for emergency physicians and even cardiologists. The differential diagnosis of elevation of the ST segment is wide including conditions with secondary of the myocardium (for exemple dissection of aortic wall), pre existing ST elevation without acute ischemia and instances with new ST with chest pain and without evidence of ischemia (for example myocarditis or pericarditis, pulmonary embolism, electrolyte imbalance, rate related repolarization changes etc) [10]. Wang described twelve conditions of mimicking STEMI (Table 1) and highlighted the electrocardiographic clues that can be used to differentiated them from AMI [11]. Some criteria can be useful to differentiate STEMI from the elevation of ST due to non ischemic etiologies (NISTE). The most sensitive is reciprocal changes, it support the diagnosis of AMI with a positive predictive value more than 90%. Reciprocal changes were not present in our case. Due to the presence of atypical ECG changes for acute PTE in our patient, AMI was considered initially in the differential diagnosis and a coronary angiogram was performed before other non invasive tests. Other evaluations like echocardiography could be helpful in this case. TTE is a readily available bedside test that can be performed in the emergency department on admission and is helpful to differentiate massive PTE and anteroseptal AMI. The ST segment changes in the ECG of this case were similar to those of previous report [12]. However, ST segment elevation is not among the usual findings associated with PE. It probably occurs due to acute right ventricular strain and elevated pressures resulting from a sub massive or massive PE [13]. Chia *et al*. [14] described ECG findings of ST segment elevation and a qs or qr pattern in 3 patients with PE in the right precordial leads those abnormalities were mostly normalized within 6 weeks due to the transient nature of ECG abnormalities.

**Table 1:** Etiologies of ST elevation according to Wang (11)

Differential Diagnosis	Pattern
Normal ST-segment elevation	Usually V1-V4 1-3 mm: male pattern, 1 mm female pattern ST segment concave
Early repolarization	ST elevation marked in V4, II III J-point notched ST segment concave Reciprocal ST depression in aVR Mild PR-segment depression
Early repolarization with persistent juvenile T wave	Young black men Coved ST elevation and TWI in midprecordial leads
Left bundle branch block	ST-T and QRS discordant ST elevation concave
Acute myopericarditis	Diffuse ST elevation with PR depression ST elevation II III and without reciprocal depression in aVL
Hyperkalemia	ST-segment downsloping
Brugada syndrome and arrhythmogenic RV cardiomyopathy	Loss of action potential in RV epicardium only Complete/partial right bundle branch block cardiomyopathy ST downsloping and saddleback shape Usually V1 and V2
Pulmonary embolism	Anteroseptal leads and associated with TWI
Prinzmetal angina	Transient ST elevations only
Post direct cardioversion	Striking transient ST (often 10 mm) elevations only
RV:right ventricular; TWI : T-wave inversion	

## Conclusion

Emergency physician must be aware of the importance to differentiate between STEMI and NISTE in patients presenting with symptoms suggestive of MI in order to avoid unsafe treatment. Chest pain is common in PE and a sensible ECG analyze can detect specific signs.

## Competing interests

The authors declare no competing interests.
